# On the relationship between tripartite motif-containing 22 single-nucleotide polymorphisms and COVID-19 infection severity

**DOI:** 10.1186/s40246-022-00394-z

**Published:** 2022-08-26

**Authors:** Nidhal Raheem Juhi Al-Kaabi, Sepideh Chodari Khameneh, Mohadeseh Montazeri, Mahsa Mardasi, Jalal Mosayebi Amroabadi, Fatemeh Sakhaee, Abolfazl Fateh

**Affiliations:** 1 Department of Biology, Science and Research Branch, Islamic Azad University, Tehran, Iran; 2 Vira Pioneers of Modern Science (VIPOMC), Tehran, Iran; 3 Tissue Engineering and Biomaterials Research Center, National Institute of Genetic, Engineering and Biotechnology (NIGEB), Tehran, Iran; 4 Department of Plant Science and Biotechnology, Faculty of Life Science and Biotechnology, Shahid Beheshti University G.C, Evin, Tehran, Iran; 5 Artificial Intelligence and Multi-omics Center (AIMOC), Stavanger, Norway; 6 Department of Mycobacteriology and Pulmonary Research, Pasteur Institute of Iran, Tehran, Iran; 7 Microbiology Research Center (MRC), Pasteur Institute of Iran, Tehran, Iran

**Keywords:** Tripartite motif-containing 22, Coronavirus disease 2019, Severe acute respiratory syndrome coronavirus 2

## Abstract

**Background:**

The tripartite motif containing (*TRIM*)-22 participates in innate immune responses and exhibits antiviral activities. The present study aimed to assess of the relationship between *TRIM22* single-nucleotide polymorphisms and clinical parameters with the coronavirus disease 2019 (COVID-19) infection severity.

**Methods:**

*TRIM22* polymorphisms (rs7113258, rs7935564, and rs1063303) were genotyped using TaqMan polymerase chain reaction (PCR) assay in 495 dead and 497 improved severe acute respiratory syndrome coronavirus 2 (SARS-CoV-2)-positive patients.

**Results:**

In this study, the frequencies of *TRIM22* rs1063303 GG, rs7935564 GG, and rs7113258 TT were significantly higher in dead patients than in improved patients, and higher viral load with low PCR Ct value was noticed in dead patients. The multivariate logistic regression analysis revealed that the lower levels of low-density lipoprotein (LDL), cholesterol, PCR Ct value, and lower 25-hydroxyvitamin D, and also higher levels of erythrocyte sedimentation rate (ESR), C-reactive protein (CRP), and *TRIM22* rs1063303 GG, rs7113258 TT, and rs3824949 GG genotypes were related to the COVID-19 infection severity.

**Conclusion:**

Our finding proved the probable relationship between the COVID-19 infection severity with the genotypes of *TRIM22* SNPs and clinical parameters. More research is required worldwide to show the association between the COVID-19 infection severity and host genetic factors.

## Introduction

In December 2019, in Wuhan, China, the severe acute respiratory syndrome coronavirus 2 (SARS-CoV-2) was the causative agent of an ongoing pandemic of coronavirus disease 2019 (COVID-19), a respiratory disease and exhibiting symptoms similar to pneumonia [1]. In addition to viral genetic variation, social, economic, and host are recognized to have effects on the incidence of COVID-19. Accordingly, the characterization of the host’s genetic factors contributing to this disease will be of great interest in prognosis and treatment [2].

Innate immunity is the first line of defense against viruses and aims to protect a host from the invasion of viruses. A part of this complex network of cells and soluble factors is each cell’s unique ability to initiate a series of intracellular responses to viral infections to limit replication and the further spread of the viruses [3]. The genome of viruses and the early products of viral replication are recognized by many pattern recognition receptors, resulting in the synthesis of type I interferons (IFNs) and activating the IFN-stimulated genes (ISGs) cascade with antiviral functions. In this regard, some members of the tripartite motif (TRIM) family are antiviral executors [4].

The members of the TRIM protein are divided into 11 families, C-I to C-XI, based on the overall domain structure; however, one group of TRIM proteins remains unclassified due to the absence of the really interesting new gene (RING) domain (e.g., TRIM14 and TRIM20). Many of these proteins have antiviral effects belonging to the C-IV family [5].

TRIM22 is involved in cell restriction of a wide variety of viruses, including human immunodeficiency virus (HIV), hepatitis B virus (HBV), hepatitis C virus (HCV), influenza A virus, encephalomyocarditis virus, and herpes simplex virus, and also *TRIM22* single-nucleotide polymorphisms (SNPs) have antiviral effect in several viral infections such as measles, HBV, HCV, and HIV [6, 7].

Many reports have indicated that SNPs might play critical roles in the COVID-19 infection severity, including polymorphisms located around the interferon lambda 3/4 *(IFNL3/IFNL4)* region, interferon-induced transmembrane protein-3, the transmembrane protease serine 2 (TMPRSS2), and the angiotensin-converting enzyme 2 (ACE2) [8, 9].

However, there are no data on the impact of *TRIM22* SNPs on the COVID-19 infection in Iran. Accordingly, the present study aimed at evaluating the effects of three *TRIM22* SNPs on the COVID-19 infection recovery and severity.

## Materials and methods

### Study population

The present study was performed on 992 COVID-19 patients with the same ethnic group at the Pasteur Institute of Iran (PII) from July 2021 to October 2021. In this study, reverse transcriptase real-time polymerase chain reaction (rtReal Time-PCR) from nasopharyngeal or oropharyngeal swab samples was used to detect SARS-CoV-2. There was no underlying disease such as chronic obstructive pulmonary disease, cystic fibrosis, asthma, obesity, diabetes, liver disease, cancer, HIV, heart and chronic kidney disease, pregnancy, and others.

About 10 ml of blood samples was taken from the patients, and peripheral blood mononuclear cells (PBMCs) were extracted by Ficoll (Ficoll-Paque PLUS, GE Healthcare, USA) density gradient centrifugation and stored at − 70 °C. The laboratory parameters including 25-hydroxyvitamin D, C-reactive protein (CRP), white blood cells (WBC), hemoglobin, erythrocyte sedimentation rate (ESR), fasting blood glucose (FBS), low-density lipoprotein (LDL), cholesterol, triglyceride (TG), high-density lipoprotein (HDL), alanine aminotransferase (ALT), aspartate aminotransferase (AST), alkaline phosphatase (ALP), blood urea nitrogen (BUN), serum creatinine, uric acid, triiodothyronine (T3), thyroxine (T4), thyroid-stimulating hormone (TSH), platelets, and real-time PCR Ct values were extracted from the patients’ records.

### DNA extraction and *TRIM22* SNPs genotyping

According to the manufacturer's instructions, the genomic DNA of the COVID-19 patients was extracted from PBMCs using the High Pure PCR Template Preparation Kit (Roche Diagnostics Deutschland GmbH, Mannheim, Germany). Three SNPs of *TRIM22* (rs1063303, rs7935564, and rs7113258) were genotyped by TaqMan® real-time allelic discrimination method, as previously described [7]. The selection criteria for *TRIM22* polymorphisms included SNPs found in a putative regulatory area, minor allelic frequency (MAF) larger than 20% for Asia and Iran, and tagSNP selection based on linkage disequilibrium (LD) > 0.8 [7, 10].

### Statistical analysis

All data were analyzed using the statistical software IBM SPSS for Windows version 22.0. (SPSS. Inc., Chicago, IL, USA). The Shapiro–Wilk test was used to evaluate the normality of the ordinal variables. Pearson's Chi-square and Mann–Whitney U tests were also used to evaluate quantitative and continuous variables, respectively. A multivariate logistic regression analysis was performed using the Hosmer–Lemeshow test to analyze the correlation between COVID19 resistance and some risk factors for susceptibility. A two-tailed *P* < 0.05 was set as the significance level. The area under the receiver-operating characteristic curve (AUC-ROC) analysis was used to assess the effect of *TRIM22* SNPs on resistance and susceptibility to COVID19. The correlation between COVID-19 mortality and *TRIM22* SNPs and haplotypes analysis were investigated using the SNPStats software inheritance mode analysis. The best-fit model for each SNP was determined using the Akaike information criterion (AIC) and the Bayesian information criterion (BIC). The Chi-square test was used to estimate the Hardy–Weinberg equilibrium (HWE) for genotype distributions [11].

## Results

### Baseline characteristics of COVID-19 patients

The present study included 992 COVID-19 patients assigned into two groups: dead patients (n = 495) and improved patients (n = 497). The mean ages of dead and improved patients were 62.6 ± 11.9 and 51.1 ± 11.8 years, respectively. The severity of COVID-19 infection was significantly associated with lower levels of cholesterol (*P* < 0.001), TG (*P* = 0.025), LDL (*P* < 0.001), 25-hydroxyvitamin D (*P* < 0.001), and low real-time PCR Ct value (*P* = 0.010) and high levels of ESR (*P* < 0.001) and CRP (*P* < 0.001). Table 1 shows the patient's laboratory and clinical features.

**Table 1:** Comparison laboratory parameters between dead and improved patients infected with COVID-19

Variables	dead patients (*n* = 495)	Improved patients (*n* = 497)	*P*-value
Mean age ± SD	62.6 ± 11.9	51.1 ± 11.8	0.515
Gender (male/female)	259/236 (52.3/47.7%)	270/227 (54.3/45.7%)	0.527
ALT, IU/L (mean ± SD) (Reference range: 5–40)	32.6 ± 19.5	31.2 ± 20.1	0.144
AST, IU/L (mean ± SD) (Reference range: 5–40)	33.9 ± 18.2	30.8 ± 17.4	0.064
ALP, IU/L (mean ± SD) (Reference range: up to 306)	178.7 ± 81.4	163.9 ± 79.4	0.073
Cholesterol, mg/dL (mean ± SD) (Reference range: 50–200)	116.1 ± 46.4	128.5 ± 51.2	< 0.001*
TG, mg/dL (mean ± SD) (Reference range: 60–165)	128.7 ± 59.8	137.1 ± 61.2	0.025*
LDL, mg/dL (mean ± SD) (Reference range: up to 150)	62.3 ± 29.7	95.4 ± 48.2	< 0.001*
HDL, mg/dL (mean ± SD) (Reference range: > 40)	32.1 ± 11.9	34.4 ± 12.5	0.813
WBC, 10^9^/L (mean ± SD) (Reference range: 4000–10,000)	7553.0 ± 2680.1	7773.5 ± 2865.9	0.245
CRP, mg/L (mean ± SD) (Reference range: < 10 mg/L Negative)	70.1 ± 21.4	51.9 ± 19.2	< 0.001*
ESR, mm/1st h (mean ± SD) (Reference range: 0–15)	56.3 ± 15.7	45.9 ± 15.1	< 0.001*
FBS, mg/dL (mean ± SD) (Reference range: 70–100)	106.8 ± 41.5	107.2 ± 42.1	0.806
Platelets × 1000/cumm (mean ± SD) (Reference range: 140,000–400,000)	185 ± 77	183 ± 68	0.577
T3, ng/dL (mean ± SD) (Reference range: 2.3–4.2)	3.5 ± 1.6	2.7 ± 1.3	0.066
T4, mcg/dL (mean ± SD) (Reference range: 5.6–13.7)	9.1 ± 6.8	8.4 ± 6.3	0.128
TSH, mu/L (mean ± SD) (Reference range: 0.4–4.5)	3.4 ± 2.1	3.1 ± 1.7	0.808
Hemoglobin, g/dL (mean ± SD) (Reference range: 12–18)	14.2 ± 2.2	12.8 ± 1.5	0.378
BUN, mg/dL (mean ± SD) (Reference range: 15–45)	39.2 ± 6.2	38.1 ± 7.1	0.527
Creatinine, mg/dL (mean ± SD) (Reference range: 0.6–1.4)	1.5 ± 0.7	0.9 ± 0.3	0.107
25-hydroxy vitamin D, ng/mL (mean ± SD) (Sufficiency: 21–150)	19.4 ± 9.2	40.2 ± 12.8	< 0.001*
Real-time PCR Ct values	12.4 ± 6.2	29.1 ± 9.3	0.010*
*TRIM22* rs1063303			
CC genotype	69 (13.9%)	197 (39.6%)	< 0.001*
GC genotype	258 (52.2%)	224 (45.1%)	
GG genotype	168 (33.9%)	76 (15.3%)	
*TRIM22* rs7935564			
AA genotype	89 (18.0%)	261 (52.5%)	< 0.001*
AG genotype	159 (32.1%)	186 (37.4%)	
GG genotype	247 (49.9%)	50 (10.1%)	
*TRIM22* rs7113258			
AA genotype	109 (22.0%)	294 (59.2%)	< 0.001*
AT genotype	159 (32.1%)	151 (30.4%)	
TT genotype	227 (45.9%)	52 (10.4%)	

*ALT* alanine aminotransferase; *AST* aspartate aminotransferase; *ALP* alkaline phosphatase; *TG* triglyceride; *LDL* low-density lipoprotein; *HDL* high-density lipoprotein; *WBC* white blood cells; *CRP* C-reactive protein; *ESR* erythrocyte sedimentation rate; *FBS* fasting blood glucose; *T3* triiodothyronine; *T4* thyroxine; *TSH* Thyroid-stimulating hormone; *BUN* Blood urea nitrogen; *Ct* cycle threshold; *SD* standard deviation

*Statistically significant (< 0.05)

### Relationship between *TRIM22* SNPs and COVID-19 infection mortality

The impact of *TRIM22* SNPs on the severity of COVID-19 infection is indicated in Table 1. In brief, the severity of COVID-19 infection was significantly higher in patients with *TRIM22* rs1063303 GG, *TRIM22* rs7935564 GG, and *TRIM22* rs7113258 TT genotypes, whereas other *TRIM22* genotypes (rs1063303 CC, rs7935564 AA, and rs7113258 AA genotypes) were observed in COVID-19 improved patients. The study's findings revealed that the patients with a co-expression of the *TRIM22* rs1063303 CC, rs7935564 AA, and rs7113258 AA genotypes (39.0%) had demonstrated a better response to COVID-19 infection compared to patients having other genotypes (32.9%).

SNPStats was used to construct the *TRIM22* SNPs inheritance model (codominant, dominant, recessive, and overdominant). The best-fit inheritance model for *TRIM22* rs1063303 was codominant with the lowest AIC and BIC. The GG genotype was associated with a higher mortality rate (OR 6.31, 95% CI 4.29–9.28, *P* < 0.0001). Minor allele frequency (G) in improved, dead, and all patients was 0.38, 0.6, and 0.49, respectively (Table 2). *TRIM22* rs1063303 genotypes were compatible with HWE in all patients (*P* = 0.341).

**Table 2:** *TRIM22* rs1063303, rs7935564, and rs7113258 association with COVID-19 mortality

*TRIM22* rs1063303 C > G		Groups		OR (95% CI)	*P*-value	AIC	BIC
Model	Genotype	Improved patients	Dead patients				
Allele	C	618 (62.3%)	396 (39.9%)	–	–	–	–
	G	376 (37.7%)	594 (60.1%)	–	–	–	–
Codominant	C/C	197 (39.6%)	69 (13.9%)	1.00	< 0.0001*	1279.0	1293.7
	C/G	224 (45.1%)	258 (52.1%)	3.29 (2.37–4.56)			
	G/G	76 (15.3%)	168 (33.9%)	6.31 (4.29–9.28)			
Dominant	C/C	197 (39.6%)	69 (13.9%)	1.00	< 0.0001*	1293.0	1302.8
	C/G-G/G	300 (60.4%)	426 (86.1%)	4.05 (2.97–5.54)			
Recessive	C/C–C/G	421 (84.7%)	327 (66.1%)	1.00	< 0.0001*	1331.8	1341.6
	G/G	76 (15.3%)	168 (33.9%)	2.85 (2.09–3.87)			
Overdominant	C/C-G/G	273 (54.9%)	237 (47.9%)	1.00	0.026*	1374.3	1384.1
	C/C	224 (45.1%)	258 (52.1%)	1.33 (1.03–1.70)			
Minor allele frequency (G)		0.38	0.6	–	–	–	–

*TRIM22* rs7935564 A > G							
Allele	A	708 (71.4%)	337 (34.0%)	–	–	–	–
	G	286 (28.6%)	653 (66.0%)	–	–	–	–
Codominant	A/A	261 (52.5%)	89 (18%)	1.00	< 0.0001*	1148.3	1163.0
	A/G	186 (37.4%)	159 (32.1%)	2.51 (1.82–3.45)			
	G/G	50 (10.1%)	247 (49.9%)	14.49 (9.83–21.35)			
Dominant	A/A	261 (52.5%)	89 (18%)	1.00	< 0.0001*	1245.3	1255.1
	A/G-G/G	236 (47.5%)	406 (82%)	5.05 (3.78–6.74)			
Recessive	A/A-A/G	447 (89.9%)	248 (50.1%)	1.00	< 0.0001*	1178.9	1188.7
	G/G	50 (10.1%)	247 (49.9%)	8.90 (6.33–12.53)			
Minor allele frequency (G)		0.29	0.69	–	–	–	–

*TRIM22* rs7113258 A > T							
Allele	A	739 (74.5%)	377 (38.0%)	–	–	–	–
	T	255 (25.5%)	613 (62.0%)	–	–	–	–
Codominant	A/A	294 (59.1%)	109 (22.0%)	1.00	< 0.0001*	1174.4	1189.1
	A/T	151 (30.4%)	159 (32.1%)	2.84 (2.08–3.88)			
	T/T	52 (10.5%)	227 (45.9%)	11.77 (8.11–17.10)			
Dominant	A/A	294 (59.1%)	109 (22%)	1.00	< 0.0001*	1233.2	1243.0
	A/T-T/T	203 (40.9%)	386 (78%)	5.13 (3.88–6.77)			
Recessive	A/A-A/T	445 (89.5%)	268 (54.1%)	1.00	< 0.0001*	1216.4	1226.2
	T/T	52 (10.5%)	227 (45.9%)	7.25 (5.17–10.16)			
Minor allele frequency (T)		0.26	0.62	–	–	–	–

*OR* odds ratios; *CI* confidence intervals; *AIC* Akaike information criterion; *BIC* Bayesian information criterion

For *TRIM22* rs7935564, the best-fitting inheritance model was codominant. A higher risk of death was associated with the GG genotype (OR 14.49, 95% CI 9.83–21.35, *P* < 0.0001). MAF (T-allele) in improved, dead, and all patients was 0.29, 0.66, and 0.47, respectively (Table 2). *TRIM22* rs7935564 genotypes were compatible with HWE (*P* = 0.062).

The best-fit inheritance model for *TRIM22* rs7113258 was codominant with the lowest AIC and BIC. The TT genotype was associated with a higher mortality rate (OR 11.77, 95% CI 8.11–17.10, *P* < 0.0001). MAF (T) in improved, dead, and all patients was 0.26, 0.62, and 0.44, respectively (Table 2). *TRIM22* rs7113258 genotypes were compatible with HWE in all patients (*P* = 0.221).

The findings of the haplotype analysis are shown in Table 3. According to our findings, the GGT haplotype was more common in the dead than in the improved patients (OR = 6.83, 95%CI = 4.87–9.59, *P* < 0.0001). Furthermore, the GGA haplotype raised the risk of mortality by OR = 6.34 (95% CI = 3.89–10.34, *P* < 0.0001). In addition, CGT (OR = 4.29, 95% CI = 2.59–7.13, *P* < 0.0001), GAT (OR = 3.02, 95% CI = 1.92–4.87, *P* < 0.0001), and CAT (OR = 2.10, 95% CI = 1.16–3.82, *P* = 0.015) haplotypes caused an increase in the risk of COVID-19 mortality.

**Table 3:** Haplotype analysis of the studied polymorphisms of *TRIM22* on COVID-19 mortality

rs1063303	rs7935564	rs7113258	Improved patients	Dead patients	OR (95% CI)	*P*-value
C	A	A	0.4054	0.1434	1.00	–
G	G	T	0.0848	0.3625	6.83 (4.87 –9.58)	< 0.0001*
G	A	A	0.1843	0.0673	0.93 (0.58 –1.51)	0.78
C	G	A	0.1153	0.0711	1.35 (0.80 –2.29)	0.26
C	G	T	0.0491	0.1269	4.29 (2.59 –7.13)	< 0.0001*
G	A	T	0.0707	0.0711	3.02 (1.92 –4.76)	< 0.0001*
G	G	A	0.0386	0.0991	6.34 (3.89 –10.34)	< 0.0001*
C	A	T	0.0520	0.0587	2.10 (1.16 –3.82)	0.015*

*OR* odds ratios; *CI* confidence intervals

*Statistically significant (< 0.05)

**Fig. 1:**
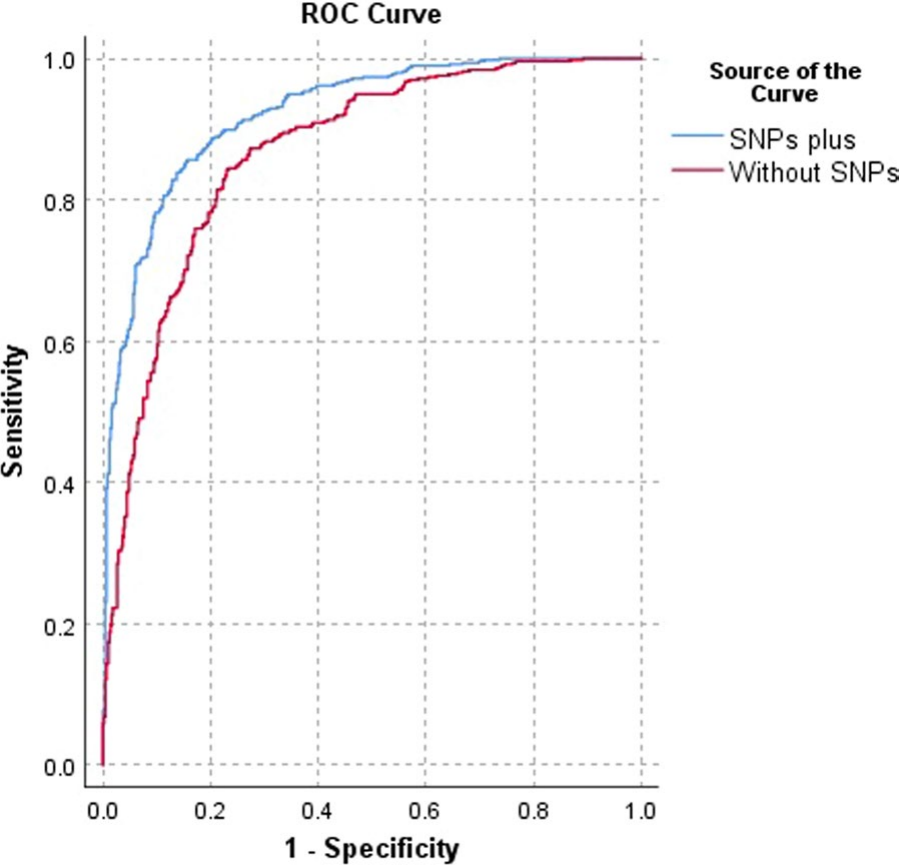
ROC curve for different predictor variable with and without the *TRIM22* SNPs genotypes

Moreover, we used logistic regression for create two different predictor variable with and without the *TRIM22* SNPs genotypes. The AUC-ROC values in with and without SNPs were 0.923 and 0.867, respectively, suggesting that host genetic factors are commonly crucial for viral infection resolution (Fig. 1).

### Factors associated with COVID-19 infection mortality

We assessed some factors associated with COVID-19 infection mortality using multivariate logistic regression analysis. In dead patients, the COVID-19 infection severity was associated with LDL (OR 0.989, 95% CI 0.981–0.998, *P* = 0.021), cholesterol (OR 1.020, 95% CI 1.003–1.038, *P* = 0.024), 25-hydroxyvitamin D (OR 1.041, 95% CI 1.026–1.056, *P* < 0.001), ESR (OR 0.958, 95% CI 0.947–0.968, *P* < 0.001), CRP (OR 0.980, 95% CI 0.972–0.988, *P* < 0.001), real-time PCR Ct values (OR 1.163, 95% CI 1.047–1.293, *P* = 0.005), *TRIM22* rs1063303 GG (OR 0.653, 95% CI 0.508–0.839, *P* = 0.001), *TRIM22* rs7935564 GG (OR 0.374, 95% CI 0.295–0.473, *P* < 0.001), and *TRIM22* rs7113258 TT (OR 0.446, 95% CI 0.353–0.565, *P* < 0.001) (Table 4).

**Table 4:** Factors associated with dead patients infected with COVID-19

Factors		
Baseline predictors	OR (95% CI)	*P*-value
LDL (mg/dL)	0.989 (0.981–0.998)	0.021*
Cholesterol, mg/dL	1.020 (1.003–1.038)	0.024*
25-Hydroxyvitamin D, (ng/Ml)	1.041 (1.026–1.056)	< 0.001*
ESR, (mm/1st h)	0.958 (0.947–0.968)	< 0.001*
CRP, (mg/L)	0.980 (0.972–0.988)	< 0.001*
Real-time PCR Ct values	1.163 (1.047–1.293)	0.005*
*TRIM22* rs1063303 (GG)	0.653 (0.508–0.839)	0.001*
*TRIM22* rs7935564 (GG)	0.374 (0.295–0.473)	< 0.001*
*TRIM22* rs7113258 (TT)	0.446 (0.353–0.565)	< 0.001*

*LDL* low-density lipoprotein; *CRP* C-reactive protein; *ESR* erythrocyte sedimentation rate; *Ct* cycle threshold; *TRIM-22* tripartite motif-22; *SD* standard deviation

*Statistically significant (< 0.05)

## Discussion

The invasion of viral infection into target cells is blocked by several host factors induced by pattern recognition receptors (PRRs) after virus recognition. Primarily, type I IFNs play a role in inducing hundreds of genes with antiviral functions. *TRIM22* is one of the genes limiting viral infections. It is constitutively expressed in epithelial cells; however, it is immediately induced by viral infection. In accordance with intracellular innate immunity effector proteins, the function of *TRIM22* is broad spectrum and is not specific to a single virus. However, several RNA and DNA viruses are restricted, and many other yet unknown viruses may be sensitive to their antiviral function. Moreover, SNPs in genes involved in the innate immune system can affect the likelihood of a clinical course of viral infection [4, 6, 12]. In other words, this was the first study to investigate whether the *TRIM22* SNPs genotypes affect the recovery or severity of COVID-19 infection.

*TRIM22* is an interferon-induced protein remarkably inhibiting the various viruses replication, including HIV-1, HCV, and HBV that is located on chromosome 11. Moreover, SNPs on *TRIM22* have been associated with several aspects of viral infections such as chronic hepatitis B infection, HIV replication, and specific antibody and cytokine levels after vaccination against measles and rubella [13–18]. Due to *TRIM22* constitutive expression in epithelial cells, its potential role in limiting the SARS-CoV-2 infection causing the currently towering COVID-19 pandemic is remarkable. However, additional experiments are recommended to determine whether *TRIM22* limits the SARS-CoV-2 replication [19].

In the present study, patients with *TRIM22* rs1063303 CC, rs7935564 AA, and rs7113258 AA genotypes had a higher rate of improved COVID-19 infection compared to others.

A significant higher prevalence of *TRIM22* rs1063303 C-allele, rs7935564 A-allele, and rs7113258 A-allele carriers was observed in dead patients than in improved patients. The allele frequency of *TRIM22* rs1063303, rs7935564, and rs7113258 in this study was 0.49, 0.44, and 0.47, respectively. This was in agreement with our previous study that we indicated the relationship between these SNPs and HCV treatment [7]. The MAF for *TRIM22* rs1063303 in European (0.52), African (0.20), Asian (0.12), other Asian (0.43), and Latin American (0.00) was reported in dbSNP the NCBI dbSNP database (https://www.ncbi.nlm.nih.gov/SNP/). For *TRIM22* rs7935564, this value was in European (0.60), African (0.49), Asian (0.18), South Asian (0.52), and Latin American (0.53). Also, for *TRIM22* rs7113258, MAF was in European (0.18), African (0.32), Asian (0.36), East Asian (0.41), and Latin American (0.32). Totally, we observed large differences in the frequency of MAF of these SNPs among the various ethnic groups.

GGT and GGA haplotypes had more association with the risk of COVID-19 mortality than other haplotypes. Most patients with COVID-19 who had GGT and GGA haplotypes were affected by the severe form of the disease.

The rs7113258 on *TRIM22* is located in 3′ untranslated region (UTR) and could be the binding site for miRNAs. Accordingly, it may have a regulatory effect on the expression of the *TRIM22* gene [20]. By binding to levels in the 3′UTR region, miRNAs can function as the negative regulators of gene expression [21]. It is documented that the *TRIM22* rs7113258 A allele generates putative target sites for many miRNAs such as hsa-miR-4495, hsa-miR-3148, and hsa-miR-3668; however, the *TRIM22* rs7113258 T allele damages these target sites and generates other miRNAs, including hsa-miR-4678 and hsa-miR-3177-5p. Therefore, the differences in miRNAs binding between the *TRIM22* rs7113258 genotypes may be associated with the observed correlation [7, 10].

Furthermore, SNPs in *TRIM22* are involved in several aspects of viral infections, including the replication of HIV, HBV infection, and HCV infection [7, 10, 17]. In this regard, *TRIM22* rs1063303 G > C makes amino acids exchange from arginine to threonine at position 242 of the TRIM22 protein. The *TRIM22* rs1063303 GG variant is correlated with the adverse effect of increasing *TRIM22* expression and reducing TRIM22’s antiviral activity [22]. In this respect, *TRIM22* overexpression was negatively correlated with HCV and HIV-1 viral load [15, 23]. On the other hand, *TRIM22* gene silencing increased HIV1 infection in target cells [15]. Moreover, the *TRIM22* rs1063303 GG genotype is also correlated with more efficient replication of HIV1 [17]. In this study, the *TRIM22* rs1063303 GG genotype was correlated with the COVID-19 infection severity with a high viral load. There is a possibility that the rs1063303 GG genotype may interfere with the regulation of HCV replication and promote a more robust inflammatory response, which could increase the infection severity more efficiently, as reported in HCV infection [10]. Further, the replication of HIV-1 was more effective in PBMCs from patients with *TRIM22* rs7935564 G allele than from patients with *TRIM22* rs7935564 A allele [17]. In the present study, *TRIM22* rs7935564 GG was associated with the COVID-19 infection severity.

The *TRIM22* rs1063303 GG, rs7935564 GG, and rs7113258 TT genotypes were strongly associated with the severity of COVID-19 infection. Using the ROC curve analysis, the most powerful predictive factor of the COVID-19 infection severity was *TRIM22* rs7935564 (GG). To the best of our knowledge, there is no report on the genotypes frequencies of *TRIM22* SNPs related to COVID-19 infection. These genotypes of *TRIM22* SNPs may be an independent predictor of the COVID-19 infection severity.

In our study, the high levels of CRP and ESR and the low levels of LDL and real-time PCR Ct were significantly associated with the COVID-19 infection severity. These results were in agreement with previous studies, indicating that disease severity was correlated with the lower levels of total LDL and cholesterol [24]. Lowering cellular cholesterol can increase cholesterol uptake from the bloodstream, leading to lower serum HDL and LDL cholesterol levels. This may lead to the upregulation of lipoprotein receptors, especially scavenger receptor class B type 1, thereby enhancing cholesterol uptake into the plasma membrane and SARS-CoV-2 infection rates [25].

T-regulatory lymphocytes (Tregs) are the primary defense against uncontrolled inflammation, and common viral infections. The Treg levels are low in many patients infected with SARS-CoV-2 and may be increased with 25-hydroxyvitamin D supplementation. The low levels of 25-hydroxyvitamin D are associated with an increased inflammatory cytokine and a significantly increased risk of pneumonia and viral upper respiratory tract infections. Moreover, 25-hydroxyvitamin D deficiency is correlated with an increase in thrombotic episodes commonly observed with COVID-19 [26].

In the present study, low Ct of rtRT-PCR was found in dead patients. Some studies have demonstrated that low PCR-Ct levels increase the risk of hospitalization and death in the intensive care unit. A correlation between viral load and the disease severity, as determined by PCR-Ct measurements, was also suggested [9].

One of the limitations of this study was the lack of data on survey correlations between ethnics and these SNPs on *TRIM22*. Another limitation was the PCR-Ct value; however, this relationship has not yet been fully standardized and quantified.

In conclusion, the present study's finding revealed a strong correlation between the lower levels of real-time PCR Ct values, 25-hydroxyvitamin D, LDL, cholesterol, and higher levels of ESR and CRP the severity of COVID-19 infection. We also demonstrated that patients with *TRIM22* rs1063303 GG, rs7935564 GG, and rs7113258 TT were exposed to more severe COVID-19 infection compared to patients with other genotypes. Further experiments are recommended to confirm the findings.

## Data Availability

All data generated or analyzed during this study are included in this published article.
